# The Bidirectional Relationship Between the Gut Microbiome and Mental Health: A Comprehensive Review

**DOI:** 10.7759/cureus.80810

**Published:** 2025-03-19

**Authors:** Kanchanbala Rathore, Neha Shukla, Sunil Naik, Kumar Sambhav, Kiran Dange, Dhrubajyoti Bhuyan, Quazi Mohammad Imranul Haq

**Affiliations:** 1 Department of Ophthalmology, Symbiosis Medical College for Women, Pune, IND; 2 Department of Dermatology, Venereology, and Leprosy, Gajra Raja Medical College, Gwalior, IND; 3 Department of Physiology, All India Institute of Medical Sciences, Mangalagiri, Mangalagiri, IND; 4 Department of Anatomy, All India Institute of Medical Sciences, Jodhpur, Jodhpur, IND; 5 Department of Dermatology, Venereology, and Leprosy, Byramjee Jeejeebhoy Government Medical College and Sassoon General Hospital, Maharashtra University of Health Sciences, Pune, IND; 6 Department of Psychiatry, Assam Medical College and Hospital, Dibrugarh, IND; 7 Department of Biological Sciences and Chemistry, College of Arts and Sciences, University of Nizwa, Nizwa, OMN

**Keywords:** dysbiosis, gut-brain axis, gut microbiome, mental health, neuroinflammation, probiotics, short-chain fatty acids

## Abstract

The gut microbiome plays a fundamental role in mental health, influencing mood, cognition, and emotional regulation through the gut-brain axis. This bidirectional communication system connects the gastrointestinal and CNS, facilitated by microbial metabolites, neurotransmitters, and immune interactions. Recent research highlights the association between gut dysbiosis and psychiatric disorders, including anxiety, depression, and stress-related conditions. Key findings indicate that altered microbial diversity, decreased short-chain fatty acid (SCFA) production, and increased neuroinflammation contribute to mental health disturbances. This paper explores the mechanism linking the gut microbiome to brain function, including microbial neurotransmitter synthesis, vagus nerve signaling, and hypothalamic-pituitary-adrenal (HPA) axis modulation. Additionally, it evaluates the potential of microbiome-targeted interventions, such as probiotics, prebiotics, dietary modifications, and fecal microbiota transplantation (FMT), in alleviating psychiatric symptoms. Microbiome sequencing and bioinformatics advances further support the development of personalized microbiome-based mental health interventions. Despite promising evidence, challenges such as inter-individual variability, methodological inconsistencies, and the need for longitudinal studies remain. Future research should focus on standardizing microbiome assessment techniques and optimizing therapeutic applications. Integrating precision psychiatry with microbiome-based diagnostics holds immense potential in transforming mental health treatment.

## Introduction and background

The gut microbiome is trillions of microorganisms, viruses, fungi, and archaea living in the gastrointestinal tract that maintain human health. Traditionally, these microbes have been understood to play roles in digestion and immune homeostasis. However, recent research has expanded our understanding, revealing a significant connection between the gut microbiome and brain function, often referred to as the gut-brain axis (GBA) [1].

Ever since the discovery of GBA, there has been increasing interest in the influence of the gut microbiome on mental health, especially regarding mood disorders, for instance, anxiety and depression. Gut microbiota is expected to have neuroactive potential owing to their capacity to produce major neurotransmitters, serotonin, dopamine, and gamma amino butyric acid (GABA), involved in emotional regulation [2,3]. Some strains of *Lactobacillus *and *Bifidobacterium *have verified their capability to interact with the HPA axis, modulate stress-related responses, and alleviate symptoms of depression [4]. On the contrary, disruptions in microbial diversity (termed gut dysbiosis) can develop into an inflammatory axis, impaired neurogenesis, and increased vulnerability to psychiatric illness [5].

Recent studies are using metagenomic sequencing and metabolic analyses that support compelling evidence that individuals with anxiety and depression have distinct microbial signatures compared to healthy controls [6]. Interestingly, persons with “major depressive disorder (MDD)” are also found to have lower points of beneficial “short-chain fatty acids (SCFAs),” e.g., “butyrate and acetate,” of microbiome-consequent metabolites that play a significant role in mood regulation [7]. Moreover, co-morbid psychiatric conditions of sleep disturbances have a relationship with gut microbiota composition in association with alterations of brain function [8].

With these emerging findings, there is a clear and present need to investigate the mechanisms contributing to the gut microbiome's interaction with mental health outcomes, particularly psychiatric disorders. Even though we do not fully understand the precise causal pathways, it is increasingly recognized that therapeutic intervention is possible now that microbial research has advanced so far [9]. Because the “gut microbiome” is known to have a part in mental health, it is crucial to understand its role to be able to develop novel diagnostic and treatment strategies for psychiatric disorders. Further development of this field demands integrated research between microbiology, neuroscience, and clinical psychiatry to explain the therapeutic benefits of microbiome-focused treatments.

**Figure 1:**
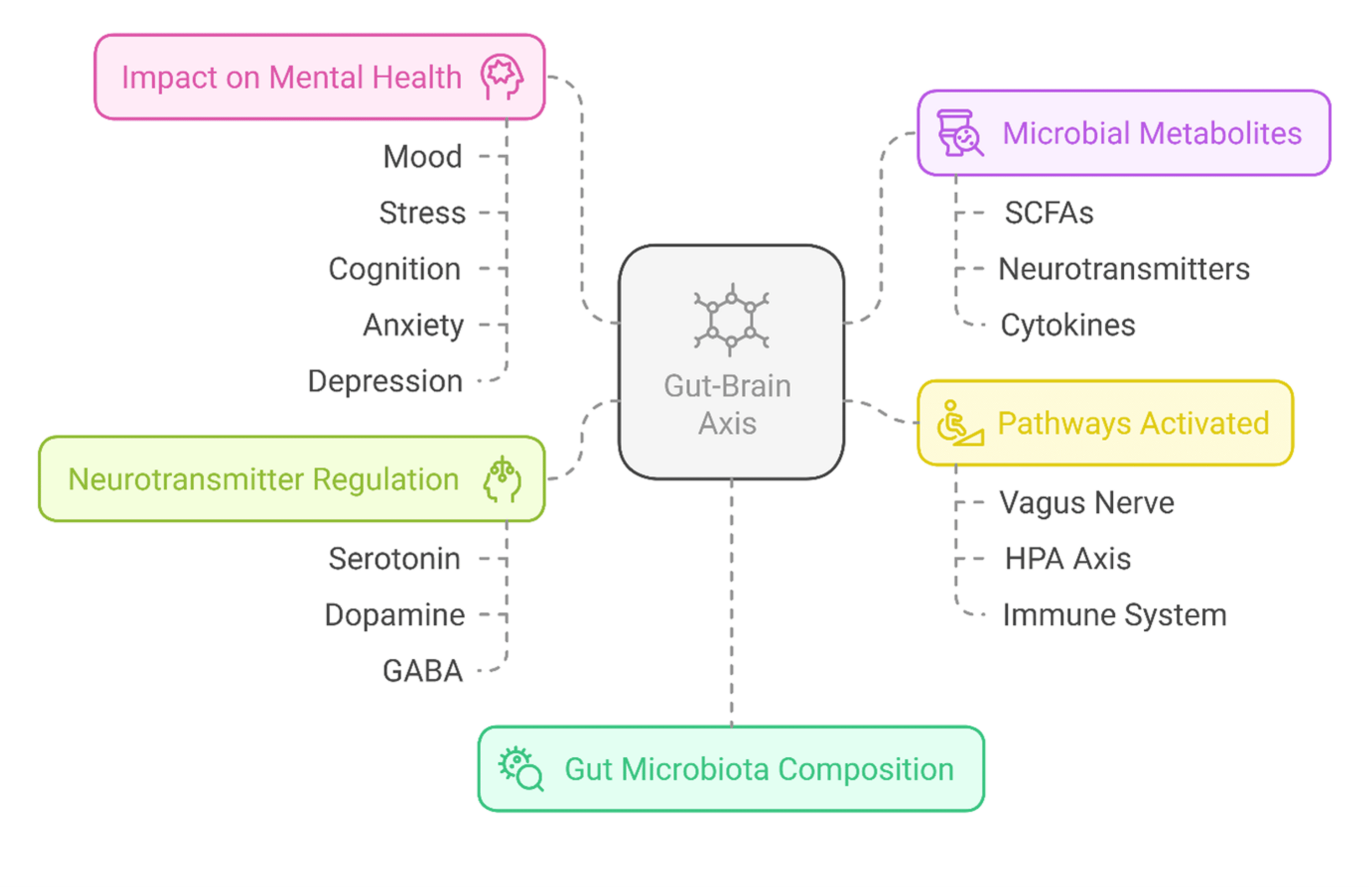
The gut-brain axis mechanism Credit: Image created by the authors SCFAs: short-chain fatty acids; GABA: gamma amino butyric acid; HPA: hypothalamic-pituitary-adrenal

As shown in Figure 1, gut microbiota impact on mental health can be achieved through modulation of neuroinflammation processes, as well as synthesis of neurotransmitters and immune responses. Understanding microbiome-targeted intervention is essential to understanding how the microbiome may present new therapeutic opportunities in the handling of neuropsychiatric ailments.

Objectives of the review

In this review, the existing research on the gut microbiome’s effect on mental well-being is synthesized to explain how the composition and variety of microbes in the gut relate to mood, cognition, and emotional regulation. In addition, it elaborates on molecular mechanisms of the GBA, for example, microbial neurotransmitter synthesis activity, vagus nerve signaling, and immune-modulating components. Finally, this review highlights the potential therapeutic effects of microbiome-targeted interventions such as probiotics, prebiotics, dietary modifications, and fecal microbiota transplantation. These interventions have shown promise in improving mental health, particularly in treating psychiatric disorders like depression, anxiety, and stress-related conditions [7,8]. New treatment strategies emerging from this research include personalized microbiome-based therapies that tailor interventions to individual microbial profiles, optimizing their effectiveness in managing mood disorders and cognitive dysfunctions.

## Review

The gut microbiome: composition and purpose

Microbiome Diversity and Key Bacterial Taxa

The gut microbiome is a multifaceted ecosystem consisting of a huge quantity of microorganisms (trillions of bacteria, viruses, fungi, and archaea). Of these, bacteria represent the most functionally important group, having roles in digestion, immune function, and neurological function. However, the human gut microbiome is predominantly comprised of four major bacterial phyla that include *Firmicutes*, *Bacteroidetes*, *Actinobacteria*, and *Proteobacteria*. The diversity of gut microbiota, determined by the relative abundance of different bacterial phyla, is essential for maintaining gut homeostasis and overall health [10].

The human gut can be characterized as having the richest phyla being “*Firmicutes *and *Bacteroidetes*,” summing to over 90% of the microbial population. Types such as *Lactobacillus *and *Clostridium *(*Firmicutes*) are mainly engaged in the fermentation of dietary fiber to yield important “SCFAs like butyrate, acetate, and propionate.” These metabolites contribute to the gut barrier integrity and their reflective effects on brain roles mediated through the GBA [11]. Polysaccharide breakdown and immune modulation are carried out by *Bacteroidetes*, including *Bacteroides *and *Prevotella*. The *Firmicutes *to *Bacteroidetes *ratio (F/B ratio) is often considered a marker for gut health, with dysbiosis associated with the onset of metabolic disorders, immune system disconcertions, and neuropsychiatric conditions [12].

Though less abundant, *Actinobacteria *are very important for gut health. Among them, *Bifidobacterium *is a phylum whose probiotic effects show the ability to lessen anxiety and mood disorders associated with high serotonin production and low gut inflammation [13]. *Bifidobacterium *has a beneficial role in mental health, as its modulation of the GBA and neuroinflammatory pathways have been shown to have.

Conversely, *Proteobacteria *(with pathogenic genera including *Escherichia *and *Salmonella*) are generally linked with gut dysbiosis and inflammatory response. Individuals with psychiatric diseases, such as MDD and generalized anxiety disorder (GAD), have shown a *Proteobacteria *overrepresentation that may underlie mental factors and contribute to negative psychosocial course [14].

To have optimal mental health, a diverse gut microbiome must be maintained. It has also been shown that reduced microbial diversity is associated with increased stress susceptibility and anxiety as well as depressive disorders [15]. This relationship is due to microbial metabolites interacting with the CNS, as well as modulating the immune response and regulating neurotransmitter levels.

Metabolic Pathways and Microbial Metabolites

It is widely accepted that the metabolic activity of the gut microbiome has a great effect on host physiology. Among the various microbial metabolites that are crucial for maintaining gut-brain homeostasis, some of the most important include SCFAs, gut-derived neurotransmitters, and inflammatory mediators.

Short-Chain Fatty Acids and Their Role in Mental Health

Bacterial fermentation of dietary fiber in the colon produces SCFAs, of which butyrate, acetate, and propionate are the primary ones. It has been studied extensively that these metabolites have neuroprotective as well as anti-inflammatory properties. Particularly, butyrate is known to enhance the gut barrier, reduce inflammation systemically, and mediate gene expression in the brain via inhibition of histone deacetylase (HDAC) [16]. Additionally, butyrate increases “brain-derived neurotrophic factor (BDNF) synthesis,” which is a key controller of neurogenesis and also enhances the process of neurogenesis along with improving cognitive function.

Modulation of the activity of the vagus nerve, which transmits signals from the gut to the brain, is how SCFAs influence the GBA [16]. It is mediated by activating specific receptors on vagal afferent fibers that respond to SCFAs, like butyrate. SCFAs can affect neural signaling pathways involving the vagus nerve that in turn influence brain function mainly in regions concerning mood regulation, stress response, and cognitive function [17]. Achieving these therapeutic effects may require targeted modulation of SCFA concentrations, particularly butyrate, which has shown the most potent anti-inflammatory and neuroprotective properties. Maintaining the balance of luminal butyrate is crucial for regulating immune function and enabling effective gut-brain communication, ultimately promoting healthy mental well-being [17].

**Figure 2:**
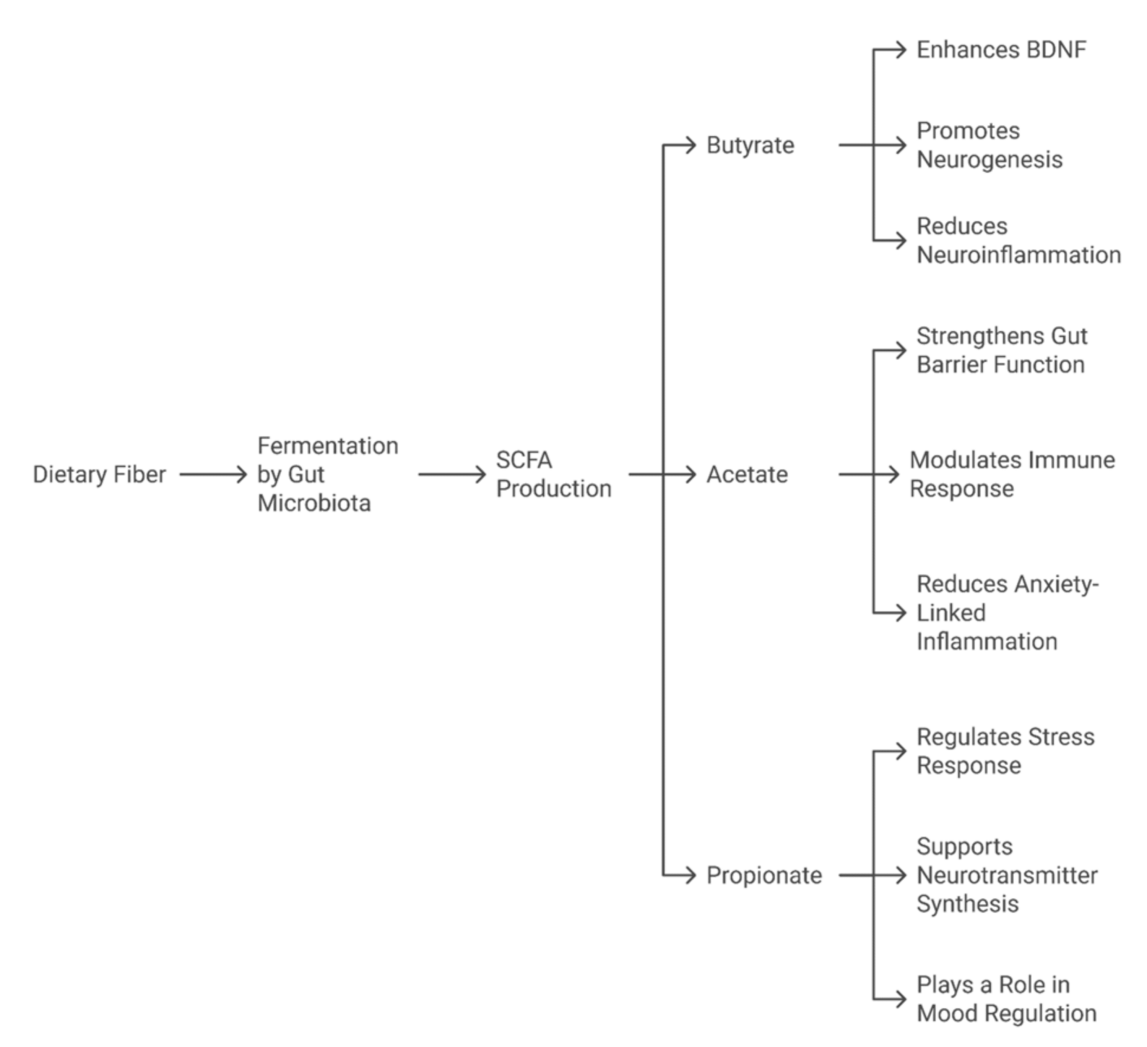
Role of SCFAs in neuroprotection Credit: Image created by the authors SCFA: short-chain fatty acid; BDNF: brain-derived neurotrophic factor

Figure 2 depicts the mechanisms by which SCFAs help the brain function and remain mentally fit. These pathways have therapeutic potential for dietary modification, probiotic microbes, or prebiotics in the treatment of mental health disorders.

Gut-Derived Neurotransmitters: Serotonin, Dopamine, and Gamma Amino Butyric Acid

In addition to SCFAs, the gut microbiota also produce key neurotransmitters that directly impact brain function, further strengthening the gut-brain connection. The synthesis of neurotransmitters by the gut microbiome is one of the most compelling aspects of the gut microbiome’s influence on mental health. “Enterochromaffin cells in the gut” produce approximately 90% of the body’s serotonin in response to microbial metabolites [18]. Serotonin is a very important neurotransmitter in the association between mood, sleep, and emotional stability. *Lactobacillus *and *Bifidobacterium *gut bacteria increase the availability of tryptophan, the precursor of serotonin, and thereby enhance its biosynthesis [13,16].

Gut microbes also produce dopamine, the neurotransmitter that is linked to mood, pleasure and reward, motivation, and cognitive function. Key contributors to dopamine synthesis have been identified as *Enterococcus *and *Bacillus *species, and disruptions in these microbial populations have been linked with mood ailments such as depression and anhedonia [19].

In addition to synthesizing catecholamines, gut bacteria also synthesize “gamma-aminobutyric acid (GABA),” the main repressive neurotransmitter in the CNS, and this phenomenon has also been observed in bacteria belonging to the species *Bacteroides *and *Lactobacillus *[20]. GABA has an important role in reducing neuronal excitability and promoting relaxation. Alterations in gut microbial composition can affect GABA production, potentially influencing the development of anxiety and stress-related disorders.

Impact of Microbial Dysbiosis on Neuroinflammation

Recently, it has been recognized that increased levels of neuroinflammation are associated with a disproportion in our gut’s microbial variety, or "gut dysbiosis," and linked to multiple psychiatric disorders. Increasing intestinal permeability, known as "leaky gut," means that bacterial endotoxins like lipopolysaccharide (LPS) will be able to enter the bloodstream. This leads to systemic inflammation that can cross-communicate with the brain, activating microglia and leading to neuroinflammation - a key feature in mood disorders such as depression and anxiety [18].

The imbalance between proinflammatory and anti-inflammatory cytokines in response to chronic inflammation induced by gut dysbiosis has been reported to exacerbate mood disturbance. In persons who have MDD and bipolar disorder, for instance, elevated proinflammatory cytokines, including interleukin-6 (IL-6) and tumor necrosis factor-alpha (TNF-α), have been detected [16], suggesting that gut-derived inflammation has a part to play in mental health conditions.

Moreover, sleep disturbances are also associated with gut dysbiosis and also lead to psychiatric ailments. It has been demonstrated that people with insomnia and other sleep disorders have a unique microbial profile, with less microbial diversity and different SCFA production. This implies that the gut microbiome could also be a hopeful therapeutic target for sleep pathology-related psychiatric conditions [13].

The Gut-Brain Axis: Mechanisms of Interaction

The GBA is a bidirectional communication system between the gut and the CNS using neural, endocrine, immune, and metabolic pathways [18]. This is a complex system that provides the control of diverse physiological and psychological processes (mood, cognition, and stress response) [19]. Studies in recent times have shown that gut microbiota influences these pathways and has a great impact on mental health and neuropsychiatric disorders [20].

Neural Pathways and Neurotransmitters

The vagus nerve, a primary conduit connecting the gut and the brain, plays a crucial role in transmitting neural signals as part of the GBA. The signals from the vagus nerve are transmitted to the brainstem, making gut microbiota selectively modulate emotional and cognitive functions. From microbial metabolites, or SCFAs and neurotransmitters like serotonin and GABA, they modulate receptors on the vagal afferent fibers. Through such enzymatic modification, these metabolites become active in the activation of specific signaling pathways, which eventually modify the activity of vagal nerves to affect the brain regions concerned with mood regulation, stress responses, and cognitive processes [20]. These signals are relayed to the CNS through the vagus nerve, which works as a key connection channel between the brain and the gut and affects the emotional and cognitive outcomes. This may lead to the potential therapeutic target of mood disorders via microbiota-mediated vagus nerve activation because research has indicated that vagal stimulation can depress the symptoms of anxiety and depression [21].

It is a well-known fact that the regulation of two of the key molecules in emotional stability and processing of reward, namely serotonin and dopamine, is heavily dependent on the gut microbiota. Approximately 90% of the body’s serotonin is produced by enterochromaffin cells, the primary source of serotonin in the body, and microbial metabolites are required for its production [22]. Serotonin is very important in the regulation of mood, anxiety, and emotional balance, and low serotonin levels have been linked to people with depression and anxiety disorders. *Lactobacillus *and *Bifidobacterium *increase the availability of tryptophan, the precursor of serotonin, and therefore increase serotonin production. *Enterococcus *and *Bacillus *species also contribute to dopamine synthesis, a neurotransmitter behind motivation, pleasure, and reward processing. Patients suffering from psychiatric disorders like depression and schizophrenia experience diminished motivation and pleasure (i.e., anhedonia) which have been associated with dopamine dysregulation, especially decreased dopamine synthesis [23].

Microbial neurotransmitter synthesis is increasingly recognized to have an impact on mood disorders. Changes in the composition of gut microbiota can result in an unbalance of neurotransmitter levels and, thereby, in mood instability and stress-related disorders. Based on studies, probiotic supplementation can enhance neurotransmitter homeostasis and thus ameliorate the symptoms of depression and anxiety by regulating the gut microbial populations [24].

Immune and Endocrine Contributions

Beyond direct neural communication, the gut microbiota also influences brain function through immune signaling and modulation of endocrine pathways, including the hypothalamic-pituitary-adrenal (HPA) axis, the central stress response system. Cortisol is released by the HPA axis, which in turn impacts mood, cognition, and inflammation. Chronic stress disturbs the equilibrium of gut microbiota and results in the increase of abdominal permeability and systemic inflammation, which further aggravates stress-related disorders [25].

The involvement of microbial communities in systemic inflammation, cytokine production, and the pathophysiology of depression and anxiety contributes to the consensus that depression is an inflammatory disorder. The interaction between cimetidine and leukotriene B4 leads to elevated levels of leukotriene B4, as well as increased concentrations of pro-inflammatory cytokines, such as IL-6 and TNF-α, which are associated with heightened neuroinflammation and depressive symptoms. Approximately developed technologies of prebiotics and dietary interventions can help mitigate inflammation and, by extension, improve mental well-being results [26].

Microbiome Modulation of Brain Activity

Several recent neuroimaging studies have strengthened the evidence that alterations in the microbiome are correlated with changes in the function of the brain. In addition, studies of fMRI, or functional magnetic resonance imaging of brain activity have found that people with a dysbiosis (that is a gut microbiota not in balance) have different activity of the amygdala and prefrontal cortex to people with a normal gut microbiome [18]. Specifically, a disrupted gut biota is related to greater amygdala activation and more emotional reactivity, anxiety, and stress response. On the other hand, there is often reduced activity of the prefrontal cortex, which is essential in controlling emotions as well as cognitive functions like making decisions and controlling one’s impulses [23]. Alteration of the brain activity could lead to mood dysregulation and cognitive deficits occurring in people who have gut microbiota imbalances. Corresponding to this, there is mounting evidence that microbiota affect brain function by neurochemical and inflammatory pathways [27], and changes in microbiota correlate with increased anxiety and depressive symptoms [28].

The microbiome and mental health disorders

Anxiety and Depression

Both human and animal models have been used to show a relationship between the absence of gut microbiota and the development of anxiety and depression. These ailments are related to a compact microbial variety and decreased amounts of beneficial species of bacteria like *Lactobacillus *and *Bifidobacterium *and higher amounts of pathogenic species, Proteobacteria in particular [29]. In animal models, germ-free mice exhibit heightened stress responses and anxiety-like behaviors, underscoring the role of microbiota in regulating emotional states [30].

Low levels of butyrate have been related to enlarged depressive symptoms, whereas acetate and propionate are involved in emotional regulation. Promising results have been obtained with interventions that aim to increase SCFA production by way of diet or supplementation to alleviate anxiety and depression [31].

Stress-Related Disorders

Stress and gut microbiota have been well established to one another; chronic stress can result in dysbiosis and increased intestinal permeability. This leads to a condition of “leaky gut” where bacterial endotoxins (e.g., LPS) enter circulation and initiate immune activation and neuroinflammatory responses [32].

Salivary cortisol is a very commonly used biomarker for stress, and recent studies indicate changes in gut microbiota structure are linked with cortisol hypersecretion. Holding more cortisol is associated with less microbial diversity and confirms the impact of the microbiome on stress resilience. Reducing cortisol levels and alleviating stress symptoms through targeted probiotic interventions have already been demonstrated [33], suggesting the adjunctive therapeutic potential of microbiome modulation in managing conditions like Cushing syndrome, where elevated cortisol levels are associated with stress and other related health issues.

Other Neuropsychiatric Conditions

The gut microbiota is equally important in neuropsychiatric disorders such as schizophrenia, bipolar disorder, and autism spectrum disorder (ASD). It has been shown that individuals with schizophrenia have unique gut microbial profiles, which contain an overgrowth of proinflammatory bacteria and a lack of SCFA-producing species. The microbial changes are associated with disease severity and cognitive impairment [34]. Adjunctive probiotic and dietary interventions aimed at improving gut health have been attempted in stabilizing mood disorders [35], though much more research remains to be done to determine how effective they are.

The other condition where gut microbiota alterations have been implicated is ASD. Many ASD children have gastrointestinal symptoms and associated microbial imbalances [36].

It has also started emerging that gut dysbiosis can be linked to neurodegenerative diseases like Alzheimer’s and Parkinson’s disease. In these conditions, dysbiotic microbiomes are related to increased inflammation, oxidative stress, and the accumulation of neurotoxic proteins. Therapeutic ways through microbiome-targeting approaches may be developed to affect these mechanisms [37].

Finally, the gut microbiome has wide-ranging effects on mental health by controlling neural, immune, and endocrine pathways. Increasingly, dysbiosis is seen as a contributing factor to several neuropsychiatric diseases, and microbiome-based therapeutics will enable a novel application to treat the same [38].

Therapeutic implications: microbiome-targeted interventions

It has also been determined that the “gut microbiota” might be a good location for beneficial intervention for mental health disorders. Probable supplementation and prebiotic supplementation, dietary changes, and FMT are among the strategies that may be used to modulate gut microbial composition to improve mental well-being results. These interventions [25] aim to re-establish microbial balance, increase the production of beneficial metabolites, and regulate the immune and neuroendocrine pathways involved in psychiatric conditions. For a comparative account of gut microbiome composition in a healthy individual and in a psychiatric patient where key microbial imbalances can be determined to be causally related to mental health disorders, see Table 1 below.

**Table 1:** Microbiome-targeted therapeutic approaches in mental health SCFA: short-chain fatty acid; GABA: gamma amino butyric acid; HPA: hypothalamic-pituitary-adrenal

Intervention	Mechanism of action	Key mental health effects	Supporting references
Probiotics (*Lactobacillus*, *Bifidobacterium*)	Restores beneficial bacteria, enhances serotonin and dopamine production, reduces inflammation	Improves mood, reduces anxiety and depressive symptoms, enhances stress resilience	[24-27]
Prebiotics (dietary fibers, inulin, fructooligosaccharides)	Stimulates SCFA production (butyrate, acetate, propionate), promotes gut integrity, modulates stress response	Reduces HPA-axis hyperactivity, improves cognitive function, and lowers depressive symptoms	[28-30]
Dietary modifications (Mediterranean diet, polyphenol-rich foods, fermented foods)	Increases microbial diversity, supports SCFA production, regulates immune response	Enhances cognitive performance, reduces neuroinflammation, protects against depression	[31-33]
Fecal microbiota transplantation (FMT)	Restores microbial diversity, corrects dysbiosis, and modulates neurotransmitter synthesis	Shows promise in alleviating severe depression, anxiety, and stress-related disorders	[34,35]
Psychobiotics (live bacteria with psychotropic effects)	Directly influences neurotransmitter synthesis (GABA, serotonin, dopamine), reduces neuroinflammation	Emerging treatment for mood disorders shows potential for PTSD and schizophrenia	[36,37]
SCFA-based therapies (butyrate supplementation)	Reduces neuroinflammation, enhances neurogenesis, strengthens gut-brain signaling	Improves emotional regulation, cognitive function, and memory	[38,39]

Probiotics and Prebiotics

Probiotics are live microbes that, when managed in adequate amounts, confer health assistance on the host, while prebiotics are “non-digestible dietary fibers that beneficially affect the host by selectively stimulating the development and/or action of one or an inadequate number of bacteria in the colon.” There is evidence that both probiotics and prebiotics have large psychobiotic effects on mood, anxiety, and cognitive function through the GBA [26].

Numerous clinical studies have shown probiotic supplementation to be effective in decreasing the symptoms of anxiety and depression. There are* Lactobacillus rhamnosus*, *Bifidobacterium longum*, and *Lactobacillus helveticus* strains that are known to affect cortisol levels, decrease systemic inflammation, and improve neurotransmitter production [27]. Probiotic administration was found to significantly reduce depressive symptoms at least as well as standard therapies [28], at least one randomized controlled trial (RCT) was observed in a meta-analysis.

Probiotics integration of mood disorders is based on several pathways affecting brain function and emotional regulation. The increased availability of tryptophan, the precursor to the neurotransmitters serotonin and dopamine, caused by the use of probiotics, enhances serotonin and dopamine biosynthesis and thereby stabilizes mood and improves cognitive function. Moreover, probiotics also help attenuate neuroinflammation by regulating cytokine production and reducing proinflammatory markers (IL-6 and TNF-α), which are increased in “patients suffering from depression and anxiety.” This is also a key mechanism in strengthening gut barrier integrity, preventing systemic inflammation, and immune activation in the setting of increased intestinal permeability (leaky gut), which is often associated with psychiatric disorders. The last involves the probiotics interaction with the vagus nerve, which is a main communication channel from the gut to the brain, and how this impacts the neurotransmitter signaling and the regulation of the autonomic nervous system. Prob integration can restore the homeostasis of the GBA and thereby suggest probiotics as a potential therapeutic target for mood disorders. There remain concerns regarding the standardization of probiotic formulations, dosing, and relative strain-specific effects because promising findings have been reported [29]. The focus of future research should be to find personalized probiotic therapies by considering individual microbiome profiles to optimize mental health benefits.

Diet and Lifestyle Modifications

The gut microbiome is highly susceptible to changes in the diet, which plays into mental health. Dysbiosis is caused by processed and high-fat diets and promotes the risk of psychiatric disorders [30], while nutrient-rich diets promote microbial diversity. One key function of maintaining gut-brain homeostasis and promoting mental well-being encompasses the role that key dietary components play in providing for microbiome health. Whole grains, legumes, and vegetables contain a good amount of dietary fiber, which promotes the production of “short-chain fatty acid (SCFA)” in the colon, which in turn reduces inflammation and also improves brain function by modulating neuroinflammatory pathways. Similarly, yogurt, kefir, and kimchi, which are fermented edibles, are rich in probiotic bacteria that enhance microbial diversity and the production of neurotransmitters in the brain [31], affecting “serotonin and dopamine.” Polyphenols found in berries, tea, and dark chocolate are also other important components that have “anti-inflammatory” and neuroprotective effects on the modulation of the "gut microbiota" and decrease oxidative stress. Therefore, these bioactive compounds impart cognitive function, emotional stability, and, in all, mental health resilience [32].

The gut-brain benefits of the Mediterranean diet have been thoroughly studied, given that it is rich in fiber, healthy fats, and polyphenol-rich foods. Adherence to the Mediterranean diet decreases the chances of depression and anxiety, which is supported by longitudinal studies relating to its effects on microbial composition and inflammation regulation [33]. Prolonged exercise has been reported to increase microbial diversity and SCFA levels, and loss of sleep also severely affects the gut microbiota homeostasis, which further affects stress-induced disorders [34].

Fecal Microbiota Transplantation

FMT is a new intercession protocol that entails the transmission of gut microbiota from healthy persons into patients with dysbiosis. FMT was initially developed for the handling of *Clostridium difficile* contaminations and is now being investigated for its use in psychiatric disorders [35]. However, FMT provides a promising therapeutic potential for depression and anxiety treatment by restoring gut dysbiosis, which is a common condition coexisting with mood disorders. Restoring microbial diversity is one of its primary benefits as microbial diversity often gets diminished in people with psychiatric conditions, thus restoring gut microbiome and enhancing gut health in general. Furthermore, FMT also enhances the generation of SCFAs, such as butyrate, which is vital in supporting neurogenesis and maintaining a balance of neurotransmitters, which is necessary for emotional regulation. In addition, one of the main mechanisms by which FMT might help mental health is via the regulation of inflammatory pathways and reducing neuroinflammation that is strongly associated with depressive and anxiety symptoms. FMT is a novel and emerging microbiome-targeted intervention in psychiatric treatment by influencing these biological pathways. Furthermore, preclinical studies have shown that transplantation of microbiota from men with depression induces depressive-like behaviors in germ-free mice, which reaffirms the fundamental relationship between the composition of the microbiota and the regulation of mood [36]. Although it has potential, ethical and clinical considerations exist. Before FMT is considered a standard psychiatric treatment, concerns concerning long-term safety, donor selection, and microbial stability need to be addressed. Its efficacy and safety for mental health applications are yet to be evaluated by the regulatory agencies [37].

Methodological considerations and limitations

GBA research will have the largest potential for neuropsychiatric applications if its methodological challenges are taken into account. The variability of microbiome composition across populations and differences in genetics, diet, geography, and environment renders any universal conclusions about healthy microbiome composition or normal status difficult to derive [38]. Because diet, medication use, and stress levels all act as confounding factors that intervene in creating fundamental relationships between microbiota and mental health outcomes, it is not possible to reach a definitive conclusion concerning the causal relationship between these two variables [39]. There are also sequencing techniques (e.g., 16S rRNA vs. metagenomic analysis) and a lack of standardized methodologies that reduce reproducibility [40]. However, the variability of microbiome signatures between individuals raises the possibility that rather than one-size-fits-all approaches, they may be needed for the treatment of psychiatric illnesses that are based on the microbiome [41]. Future research should move on to multi-omics (metagenomics, metabolomics, and transcriptomics) as a whole view of gut-brain interactions, improve bias and predictive tools, and develop therapeutic strategies. The lack of longitudinal studies and RCTs is a key research gap as they are needed to establish causality. Also needed is standardization of assessment techniques to ensure consistency of studies. The specific microbial biomarkers associated with psychiatric disorders in the setting of gut dysbiosis have not been identified [42]. Further refinement of the strain selection and dosage precision is needed for optimizing microbiome-targeted therapies, such as probiotics, prebiotics, and dietary interventions. Fill these gaps, and they will help to fill the gap in evidence-based microbiome interventions for personalized psychiatric care.

Future directions

Gut microbiome research has reached very rapid advancement and opened up new possibilities for personal mental health treatment, advanced sequencing technology, and microbiome-targeted pharmacological therapy. Because the composition of gut microbiota differs among individuals due to genetic, environmental, and dietary factors, a one-size fit may not work [42]. Also, hypervitaminosis A can result from overconsumption of vitamin A supplements or, more commonly, medications in animals like dogs. In the present review, it is important to emphasize vitamin A toxicity may cause different health problems like liver damage and neurological issues that may exacerbate the underlying conditions (neuroinflammation) [43]. It is important to recognize these risks as dietary or supplemental interventions as a part of an overall therapeutic strategy involving microbiome modulation and health [43]. With further advances in next-generation sequencing (NGS) and artificial intelligence (AI)-driven bioinformatics, microbial signatures that are most important for psychiatric disorders will be identified, the establishment of causal links between gut dysbiosis and mental health syndromes will be made, and novel microbial biomarkers will be developed from this for diagnosis and therapy monitoring. AI models are being used to predict treatment responses for mental health conditions like depression and anxiety, tailoring therapies to individual needs. Examples include AI-powered apps like Woebot, which offers CBT through a chatbot, and Clare & Me, an AI-based platform providing 24/7 mental health support via WhatsApp. These tools enhance mental health care but should complement, not replace, professional services. At the same time, given the emerging status of microbiota-based pharmaceuticals such as prebiotic drugs, SCFA mimetics, and microbiota modulating agents, and FMT-derived formulations and engineered bacterial consortia that were suggested to enhance the efficacy of traditional psychiatric treatments at lower side effects, they may offer significant health benefits. These would also represent enormous opportunities to transform psychiatric care in delivering microbiome-driven precision medicine as a crucial part of the mental health practice of tomorrow.

## Conclusions

The GBA significantly influences mental well-being, with growing evidence linking gut microbiota imbalances (dysbiosis) to anxiety, depression, and stress-related disorders. Dysbiosis can lead to neuroinflammation, neurotransmitter imbalances, and heightened stress responses, contributing to mood disturbances. Restoring microbial balance through probiotics, prebiotics, dietary changes, and FMT may improve emotional well-being and cognitive function. However, more rigorous, large-scale RCTs are needed to establish the efficacy and safety of these interventions. Additionally, AI-driven microbiome analysis is crucial for developing personalized microbiome-based treatments and precision psychiatry. Future studies should explore microbiome-based pharmacological therapies, such as SCFA-derived drugs and psychobiotics, to enhance traditional psychiatric treatments, while addressing current limitations in study design, sample sizes, and long-term effects.
